# Cetuximab in the treatment of metastatic mucoepidermoid carcinoma of the salivary glands: A case report and review of literature

**DOI:** 10.1186/1752-1947-2-320

**Published:** 2008-09-30

**Authors:** Salvatore Grisanti, Vito Amoroso, Michela Buglione, Anna Rosati, Roberto Gatta, Claudio Pizzocaro, Vittorio D Ferrari, Giovanni Marini

**Affiliations:** 1 Department of Medical Oncology, Azienda Spedali Civili, Brescia, Italy; 2 Department of Radiotherapy Oncology, University of Brescia, Brescia, Italy; 3 Department of Neurology, University of Brescia, Brescia, Italy; 4 Department of Nuclear Medicine, University of Brescia, Brescia, Italy

## Abstract

**Introduction:**

Patients with metastatic mucoepidermoid carcinoma of salivary glands have a poor outcome. The epidermal growth factor receptor protein is overexpressed in approximately 70% of mucoepidermoid carcinoma patients and may represent a therapeutic target. However, whether treatment with anti-epidermal growth factor receptor agents is effective is unclear and clinical trials are difficult due to the rarity of the disease. Here we assessed the activity of cetuximab in mucoepidermoid carcinoma on a molecular basis.

**Case presentation:**

We present the case of a 40-year old Caucasian man with a mucoepidermoid carcinoma of the major salivary glands who developed distant bone and visceral metastases despite platinum-based chemotherapy. Epidermal growth factor receptor was overexpressed and fluorescence in situ hybridization analysis demonstrated a chromosome 7 polysomy. The patient was treated with the monoclonal antibody cetuximab in combination with cisplatin. After 11 doses of cetuximab, the patient developed brain metastases but evidence of response was documented at all extracranial metastatic sites.

**Conclusion:**

This case report indicates that cetuximab can be active in mucoepidermoid carcinoma and may restore sensitivity to cisplatin in platinum-treated patients. Cetuximab does not cross the blood brain barrier and may select a metastatic clone to home the central nervous system while responding at other sites.

## Introduction

Salivary gland carcinomas (SGC) are rare neoplasms that account for less than 1% of all human cancers. Among SGCs, the mucoepidermoid carcinoma (MEC) is the most common primary neoplasm, representing approximately 30% of salivary malignancies. MEC is histologically characterized by a heterogeneous cellular composition, including squamoid (epidermoid), mucus producing and intermediate cells. The tumor grade is defined according to five histological features (the cystic component, neural invasion, necrosis, number of mitoses and anaplasia) and is important in classifying low, intermediate and high grade neoplasms [1]. High-grade MEC is an aggressive disease with a 5-year survival rate of 25 to 30%. Although the disease is often localized at presentation and rarely presents with metastases, MEC tends to recur locally and to metastasize. The initial treatment of MEC, regardless of grade, is essentially based on surgical resection and eventually on adjuvant radiotherapy. Due to the rarity of the disease, current literature is scarce and often reflects data from small and heterogeneous series. Thus, no guidelines are available to support the clinician's decision and the management of metastatic MEC remains challenging. Local recurrences not amenable to further loco-regional treatments and metastatic disease are treated with systemic chemotherapy. Single-agent or combination chemotherapy with cisplatin, fluorouracil and/or paclitaxel has demonstrated activity in published series but overall response rates are unsatisfactory and of short duration [2,3]. Overexpression of the human epidermal growth factor receptor (HER) family of oncoproteins, HER1/epidermal growth factor receptor (EGFR) and HER2, has been described in approximately 70% of salivary gland carcinomas including MEC and adenoid cystic carcinoma [4] but few studies have evaluated the therapeutic relevance of an anti-EGFR/HER2 strategy in these neoplasms. Here we report the case of a metastatic MEC of the major salivary glands that was refractory to platinum-containing regimens and was treated with the anti-EGFR monoclonal antibody cetuximab in combination with chemotherapy.

## Case presentation

**Figure 1:**
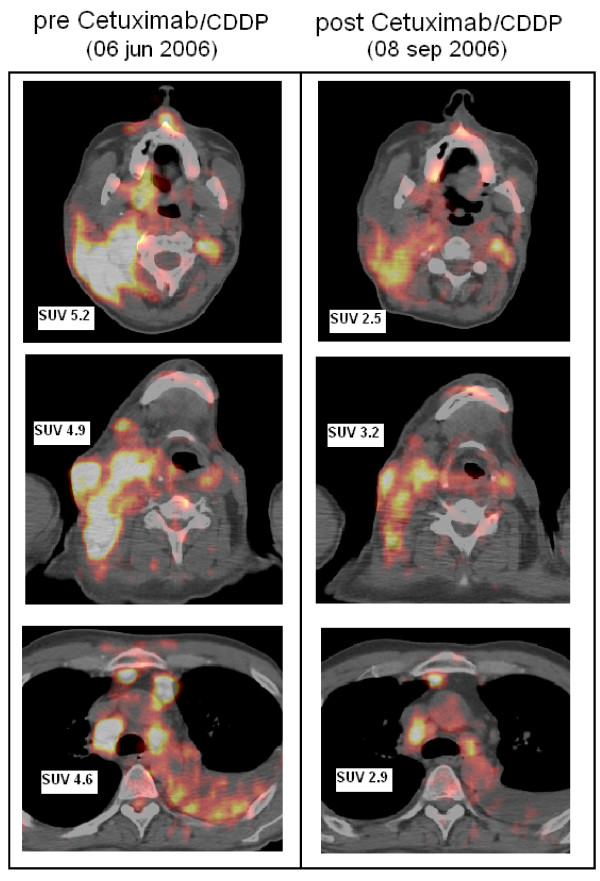
**Total body computed tomography-positron emission tomography with [^18^F]fluorodeoxyglucose before (left panel) and after (right panel) treatment.** Metabolically active neoplastic disease is located by high standardized uptake value of tracer: maximum standardized uptake value values are reported for each lesion before and after treatment.

In January 2006, a 40-year-old Caucasian man underwent a non radical resection of a high-grade MEC of the right submandibular salivary gland at another institution. Post-operative radiotherapy was not advised, and three months later, the disease progressed locally and in the cervical lymph nodes. At our institution, he was then treated with three cycles of the paclitaxel, cisplatin, fluorouracil (TPF) regimen; however, the disease progressed systemically with diffuse subcutaneous neoplastic infiltrates. He therefore received a second line chemotherapy with carboplatin and vinorelbine, but further progression occurred after two cycles. The total body [^18^F]fluorodeoxyglucose (FDG) positron emission tomography (PET) with fusion of CT-scan imaging (CT-PET) documented the onset of mediastinal adenopathies, pleural effusion and multiple bone lesions (Figure 1). Immunohistochemical analysis of the primary tumor specimen showed an intense and diffuse staining for EGFR. Cytogenetic fluorescence in situ hybridization (FISH) analysis for the EGFR gene was negative for gene amplification but demonstrated polysomy of the chromosome 7p12 (CEP7) with an average of five copies (range 3–7) of chromosome 7 per cell. Analysis of EGFR activating mutations was not performed. The patient was then treated with cetuximab (Erbitux^®^, Merck KGaA, Darmstadt, Germany), administered iv at a loading dose of 400 mg/m^2 ^over 2 hours and then at 250 mg/m^2 ^weekly in combination with cisplatin (100 mg/m^2^) every 21 days as described by Herbst *et al. *[5]. After the second cycle, a minor response was documented clinically and by CT-PET, along with the onset of a WHO grade 2 classic anti-EGFR-dependent acneiform rash; the patient continued treatment with four complete cycles of cisplatin and 11 doses of cetuximab. He also experienced WHO grade 1 paresthesias of the extremities and mild renal function impairment. Both these side effects were attributed to cisplatin.

**Figure 2:**
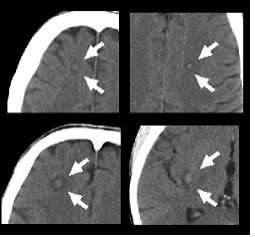
Contrast enhancement (white arrows) in brain computed tomography scan showing synchronous multiple metastases during cetuximab treatment.

In September 2006, during the treatment, the patient experienced recurrent convulsive seizures that required hospitalization. Neurologic examination was normal. An EEG showed a diffuse slowing of background activity. Rare and brief sequences of slow waves were recorded on the central regions, bilaterally. A brain CT-scan demonstrated the presence of at least five bilobar, parenchymal, metastatic lesions (Figure 2). Total-body CT-PET documented a partial response (PR > 50%) of all the evaluable extracranial metastatic lesions (Figure 1). Response was assessed according to the RECIST criteria for radiological CT imaging and to the 1999 EORTC recommendations for the use of [^18^F]fluorodeoxyglucose PET.

The patient received palliative whole brain irradiation up to 30 Gy with a daily 3 Gy fractionation and was then returned to supportive care.

In October 2006, neoplastic pleural effusion worsened and the patient died of rapidly progressive disease.

## Discussion

The epidermal growth factor receptor (EGFR) signalling pathway is involved in the physiological cell differentiation of secretory acinus and related ducts, the functional unit of salivary glands. Immunohistochemical studies demonstrated different degrees of EGFR expression in several salivary gland carcinomas, including MECs and adenoid cystic carcinomas (ACCs). In MECs, the percentage of membrane staining of EGFR is approximately 77% which is higher than in normal tissue [4]. EGFR overexpression is related to a poorer prognosis and a more aggressive behaviour of the disease but its overall prognostic value has not been completely established [3,6]. Thus, the development of anti-HER2 or anti-EGFR strategies in salivary gland carcinomas could represent a reasonable approach based on a biological rationale. In head and neck squamous cell carcinoma (HNSCC), the anti-EGFR monoclonal antibody cetuximab has been proven to prolong overall survival in combination with radiotherapy [7] and to enhance response rates in recurrent/metastatic disease in combination with cisplatin or fluorouracil [8]. Whether cetuximab can overcome platinum resistance in cisplatin-pretreated patients remains controversial [9,10]. Among predictive factors of response to anti-EGFR strategies in HNSCC, EGFR gene amplification status is predictive of sensitivity to the EGFR-tyrosine kinase inhibitor, gefitinib [11]. On the other hand, no activating mutations in the EGFR gene are associated with response to anti-EGFR strategies [12].

To date, only a few, small clinical trials have investigated the antitumor activity of anti-EGFR targeted agents in the salivary gland neoplasms. In a phase II study of cetuximab monotherapy in 22 patients with recurrent/metastatic salivary gland carcinomas, including two MECs, 11 patients had stable disease, seven of which remained progression-free for over 6 months. None of these patients displayed EGFR amplification or chromosome 7 polysomy [13]. In a recent phase II study of lapatinib, a dual inhibitor of EGFR and HER2, in recurrent/metastatic salivary gland carcinomas, including two MECs, Agulnik *et al. *demonstrated disease stabilization that lasted for more than 6 months in 36% of the patients; no objective responses were observed. None of the patients in the cohort with disease stabilization, however, were patients with MEC [14].

In our report, the patient was pretreated with two lines of platinum-containing chemotherapy with no evidence of response; a response was only observed upon treatment with cisplatin along with cetuximab. We do not know whether the response was due to cetuximab alone or due to a synergistic effect with the restoration of cisplatin sensitivity. The chromosome 7 polysomy, documented in the patient, may account for increased protein expression at the membrane level and clinical response. However, a clear genotype/phenotype correlation cannot be established on the basis of only this data. Finally, a mixed pattern of central nervous system (CNS) progression and systemic response was concomitantly observed in our patient. Such an effect is often seen in metastatic breast cancer patients treated with trastuzumab who develop CNS metastases while responding at different metastatic sites. The creation of a CNS sanctuary for cancer cells results from the inability of trastuzumab to cross the blood-brain barrier due to its relatively high molecular weight; this mechanism presumably also applies to cetuximab in different neoplastic conditions, as our case demonstrates.

## Conclusion

In conclusion, this case report indicates cetuximab function in a recurrent and metastatic MEC of the salivary glands. EGFR expression is a prerequisite for cetuximab use but EGFR gene amplification, or at least chromosome 7 polysomy, seems to be necessary for elicitation of biological activity. Our findings also emphasize the importance of CT-PET in monitoring neoplastic diseases during molecular targeted therapies. The inability of cetuximab to cross the blood-brain barrier and the consequent development of CNS metastases during treatment is a subject of concern that requires further study.

## Abbreviations

ACC: adenoid cystic carcinoma; CEP: centromeric enumeration probes; CNS: central nervous system; CT: computed tomography; EEG: electroencephalogram; EGFR: epidermal growth factor receptor; FDG: fluorodeoxyglucose; FISH: fluorescence in situ hybridization; HER: human epidermal growth factor receptor; HNSCC: head and neck squamous cell carcinoma; MEC: mucoepidermoid carcinoma; PET: positron emission tomography; SGC: salivary gland carcinoma; TPF: paclitaxel, cisplatin, fluorouracil; WHO: World Health Organization.

## Competing interests

The authors declare that they have no competing interests.

## Authors' contributions

SG was involved in the conceptual design of this study, in the clinical management of the patient, and in the writing and editing of the manuscript. VA was involved in the clinical management and contributed to writing and editing of the manuscript. MB was involved in the radiation treatment of the patient. AR performed the neurological, clinical and instrumental examinations. RG was involved in the manuscript editing and provided iconographic material. CP performed and interpreted all CT-PET scan examinations. VDF was involved in the clinical management and contributed to the writing and editing of the manuscript. GM supervised the entire treatment, provided financial and administrative support and contributed in the critical reading of the manuscript. All authors read and approved the final manuscript.

## Consent

A standard institutional informed written consent for treatment was obtained from the patient at the beginning of each treatment. A separate institutional informed consent was obtained at the first visit, with regard to the treatment of personal data and use in scientific publications and with regard to protection of privacy according to Italian law. Written informed consent was obtained from the patient for publication of this case report and accompanying images. A copy of the written consent is available for review by the Editor-in-Chief of this journal.
